# Is the total score of the Hamilton Depression Rating Scale affected by side effects of SSRIs and SNRIs?

**DOI:** 10.1192/j.eurpsy.2023.43

**Published:** 2023-07-19

**Authors:** S. D. Østergaard

**Affiliations:** Aarhus University Hospital - Psychiatry, Aarhus, Denmark

## Abstract

**Abstract:**

This talk will focus on the pitfalls of using multidimensional rating scales to measure the severity of depression – with particular emphasis on the Hamilton Depression Rating Scale (HDRS-17). First, the history behind the development of the HDRS-17 will be briefly covered. Second, it will be argued that the HDRS-17 measures symptoms that overlap with common antidepressant side effects (gastrointestinal dysfunction, sexual dysfunction, somatic anxiety and sleep disturbances), making it possible that side effects of antidepressant treatment are erroneously rated as symptoms of depression.

The rest of the talk will focus on the results of a recent study (1) in which we used individual-level data from antidepressant treatment trials to assess whether side effect ratings co-vary with HDRS-17 ratings. Specifically, data from all HDRS-17-rated, industry-sponsored pre- and post-marketing trials (n = 4647) comparing the serotonin and noradrenaline reuptake inhibitor, duloxetine, to placebo and/or to a selective serotonin reuptake inhibitor were pooled. Severity was assessed for side effects related to sleep, somatic anxiety, gastrointestinal function, and sexual dysfunction. Analysis of covariance was used to assess the relation between these side effects and ratings of relevant HDRS-17-derived outcome parameters. Side effects related to sleep, somatic anxiety and sexual dysfunction significantly and exclusively associated with higher scores on HDRS-17 items measuring the corresponding domains. Side effects related to gastrointestinal function associated with higher HDRS-17 item scores on all assessed domains. Treatment outcome was significantly related to side effect severity when assessed using HDRS-17-sum (beta 0.32 (0.074), p < 0.001), but not when the HDRS-6-sum-score (beta 0.035 (0.043), p = 0.415) or the depressed mood item (beta 0.007 (0.012), p = .527) were used as effect parameters. That some HDRS-17 items co-vary with common antidepressant side effects likely leads to an underestimation of antidepressant efficacy. Finally, based on data from a recent study (2), it will be argued that the Montgomery-Åsberg Depression Rating Scale is biased in the same direction as the HDRS-17 (underestimates antidepressant efficacy), albeit to a lesser extent.

**References:**

1. Hieronymus et al. Do side effects of antidepressants impact efficacy estimates based on the Hamilton Depression Rating Scale? A pooled patient-level analysis. Transl Psychiatry. 2021;11:249.

2. Hieronymus et al. The response pattern to SSRIs as assessed by the Montgomery-Åsberg Depression Rating Scale: a patient-level meta-analysis. World Psychiatry. 2022;21:472-473.

**Disclosure of Interest:**

S. D. Østergaard Shareolder of: SDØ owns/has owned units of mutual funds with stock tickers DKIGI, IAIMWC, SPIC25KL and WEKAFKI, and has owned units of exchange traded funds with stock tickers BATE, TRET, QDV5, QDVH, QDVE, SADM, IQQH, USPY, EXH2, 2B76 and EUNL., Grant / Research support from: SDØ is supported by grants from the Novo Nordisk Foundation (grant number: NNF20SA0062874), the Lundbeck Foundation (grant numbers: R358-2020-2341 and R344-2020-1073), the Danish Cancer Society (grant number: R283-A16461), the Central Denmark Region Fund for Strengthening of Health Science (grant number: 1-36-72-4-20), the Danish Agency for Digitisation Investment Fund for New Technologies (grant number 2020-6720) and Independent Research Fund Denmark (grant numbers: 7016-00048B and 2096-00055A).

